# Supplementary material for: Effect of rotating providers on chest compression performance during simulated neonatal cardiopulmonary resuscitation
Source: PLoS One. 2022 Mar 14;17(3):e0265072. doi: 10.1371/journal.pone.0265072 (PMC8920209; doi:10.1371/journal.pone.0265072)
Supplement: S1 Fig — (DOCX) [file pone.0265072.s001.docx]

**Participant Questionnaire**

Thank you for choosing to participate in our research study. The following questions will inquire about your daily lifestyle, personal characteristics and how fatigued you felt at the completion of this simulation session.

**Gender**: Male Female

**Age** (years):_____

**Weight** (lbs or kg) _______

**Height** (cm, ft or inches) ________

1. Do you smoke tobacco/nicotine products (for example: cigarettes, cigars, pipe, e-cigarette/vape)? If NO skip to question #3.
   1. YES
   2. NO
2. On average, how often have you smoked in the last month?
3. 20 cigarettes (1pack) or more a day
4. 10-19 cigarettes a day
5. 5 to 9 cigarettes a day
6. Less than 5 cigarettes a day
7. How many hours a week did you participate in any physical exercises (for example: running, jogging, swimming, cycling, cross-fit, Pilates, Yoga, strength training) in the last month? If NO skip to question #5

0 1 2 3 4 5 6 7 8 9 10 >10

1. How frequently did you rotate while performing CC’s today?
   1. Every 3 minutes
   2. Every 5 minutes
   3. Every 10 minutes
2. On a scale of 1-to-10, how fatigued were you by the end of this simulation? A score of 1 being not fatigued and 10 being extremely fatigued.

1 2 3 4 5 6 7 8 9 10
