# Supplementary material for: Effect of rotating providers on chest compression performance during simulated neonatal cardiopulmonary resuscitation
Source: PLoS One. 2022 Mar 14;17(3):e0265072. doi: 10.1371/journal.pone.0265072 (PMC8920209; doi:10.1371/journal.pone.0265072)
Supplement: S1 Table — (DOCX) [file pone.0265072.s002.docx]

**SUPPLEMENTAL TABLE**

|  | **Rotation frequency** | | | | | | | | |  |
| --- | --- | --- | --- | --- | --- | --- | --- | --- | --- | --- |
|  | **3 min** | | | **5 min** | | | **10 min** | | |  |
| **Covariates** | **n** | **Median (25-75th%)** | **Mean (SD)** | **n** | **Median (25-75th%)** | **Mean (SD)** | **n** | **Median (25-75th%)** | **Mean (SD)** | **p-value** |
| sbp 1 | 43 | **-4.0 (-10, 3.00)** | -3.4 (11.0) | 42 | **-7.0 (-15, -1.0)** | -6.4 (11.4) | 43 | **-11 (-15, 3.00)** | -5.8 (13.7) | 0.1878^a^ |
| sbp 2 | 43 | **-5.0 (-11, 1.00)** | -5.0 (10.7) | 42 | **-8.0 (-15, -1.0)** | -6.4 (12.6) | 0 | **. (., .)** | . (.) | 0.3289^b^ |
| sbp 3 | 43 | **-4.0 (-9.0, 6.00)** | -3.5 (16.1) | 0 | **. (., .)** | . (.) | 0 | **. (., .)** | . (.) | NA |
| **p-value** |  |  | 0.5419^A^ |  |  | 0.9871^B^ |  |  | 0.0085^B^ |  |
| dbp 1 | 43 | **1.00 (-2.0, 3.00)** | 1.07 (4.28) | 42 | **0.00 (-4.0, 2.00)** | -.88 (5.14) | 43 | **2.00 (-3.0, 6.00)** | -.51 (11.4) | 0.1926^a^ |
| dbp 2 | 43 | **1.00 (-2.0, 3.00)** | 0.33 (3.78) | 42 | **-1.5 (-3.0, 1.00)** | -1.8 (5.76) | 0 | **. (., .)** | . (.) | 0.0467^b^ |
| dbp 3 | 43 | **1.00 (-2.0, 4.00)** | 0.86 (4.63) | 0 | **. (., .)** | . (.) | 0 | **. (., .)** | . (.) | NA |
| **p-value** |  |  | 0.5419^A^ |  |  | 0.2501^B^ |  |  | 0.7698^B^ |  |
| hr 1 | 43 | **-5.0 (-10, 1.00)** | -5.8 (7.74) | 42 | **-7.0 (-12, 0.00)** | -6.0 (8.99) | 43 | **-9.0 (-15, -3.0)** | -12 (12.2) | 0.0408^a^ |
| hr 2 | 43 | **-6.0 (-10, 0.00)** | -5.6 (6.53) | 42 | **-8.5 (-15, -4.0)** | -9.8 (11.9) | 0 | **. (., .)** | . (.) | 0.0366^b^ |
| hr 3 | 43 | **-6.0 (-13, -1.0)** | -7.6 (9.85) | 0 | **. (., .)** | . (.) | 0 | **. (., .)** | . (.) | NA |
| **p-value** |  |  | 0.5419^A^ |  |  | 0.0220^B^ |  |  | <0.0001^B^ |  |
| o2 1 | 43 | **0.00 (-1.0, 1.00)** | 0.05 (1.68) | 41 | **0.00 (-1.0, 1.00)** | 0.17 (1.84) | 43 | **1.00 (0.00, 3.00)** | 1.16 (1.99) | 0.0222^b^ |
| o2 2 | 43 | **1.00 (0.00, 2.00)** | 0.79 (1.77) | 41 | **1.00 (-1.0, 2.00)** | 0.27 (3.95) | 0 | **. (., .)** | . (.) | 0.8206^b^ |
| o2 3 | 43 | **1.00 (0.00, 3.00)** | 1.02 (2.04) | 0 | **. (., .)** | . (.) | 0 | **. (., .)** | . (.) | NA |
| **p-value** |  |  | 0.5419^A^ |  |  | 0.8684^B^ |  |  | 0.0004^B^ |  |

**Supplemental Table 5** Differences in the individual vital sign parameter when compared from baseline to the vital signs taken after each round of CCs. Number of data points was dependent on the simulation performed.

Abbreviations: sbp, systolic blood pressure; dbp, diastolic blood pressure; hr, heart rate; o2, oxygen saturation. ^a^Kruskal-Wallis test, ^b^Wilcoxon-Mann-Whitney test, ^A^Repeated Measures ANOVA, ^B^Paired Student’s t-test
