# Supplementary material for: Effect of rotating providers on chest compression performance during simulated neonatal cardiopulmonary resuscitation
Source: PLoS One. 2022 Mar 14;17(3):e0265072. doi: 10.1371/journal.pone.0265072 (PMC8920209; doi:10.1371/journal.pone.0265072)
Supplement: Attachment — Submitted filename: Response to reviewers.docx [file pone.0265072.s003.docx]

Respected Editor and Reviewer,

Thank you for taking the time to review our work. We value your feedback and have attempted to respond to your queries to the best of my abilities. We have made the recommended edits as highlighted in the attached revision of the manuscript. Below are the responses under each comment from the journal, the editor and the reviewer.

**Requested Journal revisions:**

1. Please ensure that your manuscript meets PLOS ONE's style requirements.

- This has now been addressed.

2. Please review your reference list to ensure that it is complete and correct.

- Reference list has been updated with the most current references, this includes the Neonatal Life Support 2020 guidelines as well as the European Resuscitation Council Guidelines 2021 (citation 7 & 8).

3. Please include additional information regarding the survey or questionnaire used in the study and ensure that you have provided sufficient details that others could replicate the analyses.

- This has been now addressed in line 166. Questionnaire has also been added as supporting information in S1 Fig 1.

4. Please state whether you validated the questionnaire prior to testing on study participants. Please provide details regarding the validation group within the methods section.

- See line 166. The study questionnaire was not validated, and we have now put that as one of the study limitations in lines 316-317. In addition, we have added the questionnaire as supporting information in S1 Fig 1 for the readers.

**Response to the editors’ comments:**

- Minor revisions: 75 sessions line 106. 76 in table. Please ensure consistency.
  - Line 106 mentions what was intended in the protocol, however due to error in pairing of the participants we were left with less than 75 sessions and hence enrolled one additional participant and ended up with a total of 76 sessions (this is explained in the manuscript lines 192-195). Additionally, we have added this information in the text under figure 2 to provide more clarity.
- Table 4 headings are missing- table is not explained properly. It seems more CC are “missed” when providers are rotated out- this seems significant?
  - The table heading has been addressed, sorry for that miss earlier
  - It is true that the more CC were missed when the providers rotated out more frequently, however the number of CCs missed were not statistically significant when compared to the total number of the CC performed over the 18 and 20 minute runs. We have elaborated on this now in lines 214-217 for additional clarity.
- Page 12: “However, in the previous study, CC depth of one-third of chest diameter was defined as adequate quality, and a surrogate marker for provider fatigue was defined as four consecutive CCs below target depth.” This data is available for current study as well. Have the authors looked at these? From fig 2- it looked like CC depth was similar between groups here? How do you explain different findings/interpretation in same mannequin and relatively similar group of participants? If you are showing increased fatigue in 10min- why do you not see effect on quality/depth of CC compared to shorter durations?
  - The previous study had used the HeartStart Mrx monitor which allowed quantification of the rate, depth, chest deflection and residual leaning force. We solely used the Resusci Anne Skill Reporter device which provided composite data on the entire run, this included the average CC depth and % CC that were deep enough. We have included this piece of information now in line 255-258.
- Page 12 line 238: you report under compression by providers. Was there “over compression” ie compressing excessive depth by some providers- that could impact fatigue.
  - Over compression was not reported by the device. It only reports % compressions that were of adequate depth (>1/3 AP diameter). We have elaborated on this in lines 288-291.

**Response to reviewers’ comments:**

1. p3, line 63: "bpm" should be spelled out the first time the abbreviation is used.

- Done

2. p3: ref 7 and 8 should be updated to the more recent 2020 publications.
- Done

3. page 3, line 65: "bpm" abbreviation can be used here.
- Done

4. p5, line 94: CV should be spelled out as this is the first time the abbreviation has been used.
- Thank you, we have now addressed that in line 67.

5. p11, Table 4: Missing a title row for the respective columns (e.g. what do the 19.89 and 37.13 values for the 3 min row indicate?).
- Done, apologies for that missing information.

6. Methods/Results: The authors mention in the methods section that reporter fatigue was assessed by Likert scale. Can the authors include those results in the manuscript?
- This is shared in the last box plot on the Figure 3 (see below for reference) and mentioned in lines 211-213. Additionally, we have now included this information in the text under the figure.

7. Discussion, p13: can the authors comment if the five providers who performed chest compressions at <10% depth had a similar performance during their practice time and how they responded to the feedback? One would expect their performance to have improved during the simulation following the practice session.

-Performances during practice were not recorded. No verbal feedback was shared during practice either, but the participants were able to visually track the compression depth and hand positioning on the SimPad PLUS with Skill reporter device during this practice period. I have included this piece of information in lines 295-298.

We are hopeful that the revisions that have been made have successfully addressed the points that were made by the editor and reviewer. However, if any further clarification is needed, we will be happy to address that. We thank the team of PLOS ONE once again for considering this manuscript for publication.

Best regards,

Tavleen Sandhu, MD

Assistant Professor

Section of Neonatal-Perinatal Medicine

OUHSC Department of Pediatrics

University of Oklahoma HSC

1200 Everett Drive, ETNP 7504

Oklahoma City, OK 73104

P: (405) 271-5215 Ext. 43334

E: tavleen-sandhu@ouhsc.edu
